# Remodeling Pearson's Correlation for Functional Brain Network Estimation and Autism Spectrum Disorder Identification

**DOI:** 10.3389/fninf.2017.00055

**Published:** 2017-08-31

**Authors:** Weikai Li, Zhengxia Wang, Limei Zhang, Lishan Qiao, Dinggang Shen

**Affiliations:** ^1^College of Information Science and Engineering, Chongqing Jiaotong University Chongqing, China; ^2^School of Mathematics, Liaocheng University Liaocheng, China; ^3^Department of Radiology and BRIC, University of North Carolina at Chapel Hill Chapel Hill, NC, United States; ^4^Department of Brain and Cognitive Engineering, Korea University Seoul, South Korea

**Keywords:** functional brain network, functional magnetic resonance imaging, Pearson's correlation, sparse representation, scale-free, autism spectrum disorder

## Abstract

Functional brain network (FBN) has been becoming an increasingly important way to model the statistical dependence among neural time courses of brain, and provides effective imaging biomarkers for diagnosis of some neurological or psychological disorders. Currently, Pearson's Correlation (PC) is the simplest and most widely-used method in constructing FBNs. Despite its advantages in statistical meaning and calculated performance, the PC tends to result in a FBN with dense connections. Therefore, in practice, the PC-based FBN needs to be sparsified by removing weak (potential noisy) connections. However, such a scheme depends on a hard-threshold without enough flexibility. Different from this traditional strategy, in this paper, we propose a new approach for estimating FBNs by remodeling PC as an optimization problem, which provides a way to incorporate biological/physical priors into the FBNs. In particular, we introduce an L_1_-norm regularizer into the optimization model for obtaining a sparse solution. Compared with the hard-threshold scheme, the proposed framework gives an elegant mathematical formulation for sparsifying PC-based networks. More importantly, it provides a platform to encode other biological/physical priors into the PC-based FBNs. To further illustrate the flexibility of the proposed method, we extend the model to a weighted counterpart for learning both sparse and scale-free networks, and then conduct experiments to identify autism spectrum disorders (ASD) from normal controls (NC) based on the constructed FBNs. Consequently, we achieved an 81.52% classification accuracy which outperforms the baseline and state-of-the-art methods.

## Introduction

Autism spectrum disorder (ASD) is a neural developmental syndrome defined by the defect in social reciprocity, restricted communication, and repetitive behaviors (Lord et al., 2000; Frith and Happé, 2005; Baio, 2014; Wee et al., 2016). The prevalence rate of ASD is fast growing in the worldwide. According to the report supported by the USA Centers for Disease Control and Prevention (Baio, 2014), 1.47% of American children was marred by some forms of ASD with a nearly 30% increasing rate in the last 2 years. However, the standard ASD diagnosis methods (e.g., parent interview and participant interview) are highly based on behaviors, and symptoms of the disease (Gillberg, 1993; Lord and Jones, 2012; Segal, 2013), resulting in missing the best cure opportunity. At the same time, the measurement at the gene level (Wang et al., 2009; O'Roak et al., 2012) can benefit an early diagnosis, but it is less popular due to high costs and complexity. Recent evidences show that the unusual brain activity (Brambilla et al., 2003; Ecker et al., 2010; Lo et al., 2011; Nielsen et al., 2013) and abnormal functional disruptions in some brain regions (Allen and Courchesne, 2003; Anderson et al., 2011; Delmonte et al., 2012) such as, hippocampus and frontal region have a high correlation with ASD. Thus, it is possible to discover informative biomarkers and then help identify ASD by analyzing the activity data of brain.

Functional magnetic resonance imaging (fMRI) is currently a widely-used non-invasive technique for measuring brain activities (Brunetti et al., 2006; Kevin et al., 2008; Jin et al., 2010). However, it is hard to identify patients from normal controls (NC) by direct comparison of the fMRI data (i.e., time courses), since the spontaneous brain activities are random and asynchronous across subjects. In contrast, the functional brain network (FBN) constructed by, for example, the correlation of the time series can provide a more stable measurements for classifying different subjects (Smith et al., 2011; Sporns, 2011; Wee et al., 2012; Stam, 2014; Rosa et al., 2015). In fact, FBN identifies functional connections between brain regions, voxels, or ROIs (Horwitz, 2003), which has already been verified to be highly related to some neurological or psychological diseases such as, ASD (Theije et al., 2011; Gotts et al., 2012), mild cognitive impairment (Fan and Browndyke, 2010; Wee et al., 2012, 2014; Yu et al., 2016), Alzheimer's disease (Supekar et al., 2008; Huang et al., 2009; Liu et al., 2012) and so on.

The commonly used scheme to estimate FBNs is based on the second-order statistics that tend to work better than the high-order counterparts (Smith et al., 2011). The typical second-order estimation methods include Pearson's correlation (PC), and sparse representation (SR), etc. PC estimates FBNs by measuring the full correlation between different brain regions (ROIs^1^). The full correlation is simple, computationally efficient and statistically robust, but tends to include confounding effects from other brain regions. In contrast, the partial correlation can alleviate this problem by regressing out the potential confounding influence. However, calculating the partial correlation involves an inverse operation on the covariance matrix, which is generally ill-posed, especially when the number of time points is fewer than the number of brain regions. Therefore, regularization techniques such as, SR (with a L_1_-norm regularizer) are generally used to achieve a stable solution (Lee et al., 2011).

In this paper, we mainly focus on the PC-based methods, because we empirically found that, in our experiments, the PC-based (full correlation) methods work better than the SR-based (partial correlation) counterpart. However, the original PC scheme always results in FBNs with a dense topological structure (Fornito et al., 2016), since the BOLD signals commonly contain noises, micro head-motion (Power et al., 2013; Yan et al., 2013) and/or mind wandering (Mason et al., 2007). In practice, a threshold is commonly used to sparsify the PC-based FBNs by filtering out the noisy or weak connections. Although it is simple and effective, the threshold scheme is hard without enough flexibility. To address this problem, in this paper, we reformulate the estimation of PC network as an optimization problem, and motivated by the SR model (see Section Related Methods), we introduce an L_1_-norm regularizer for achieving a sparse solution. Different from the traditional hard-threshold scheme, the proposed method is more flexible, and can in principle incorporate any informative prior into the PC-based FBN construction. Specifically, the main contributions of this paper can be summarized as follows.

1. We propose a novel strategy to estimate PC by remodeling it in an optimization learning framework. Consequently, biological/physical priors can be incorporated more easily and naturally for constructing better PC-based FBNs.
2. We introduce an L_1_-norm regularizer into the proposed framework for estimating sparse FBNs, and further extend it to a weighted version for constructing both sparse and scale-free FBNs. These two instantiations illustrate that the proposed method is more flexible than the traditional hard-threshold scheme.
3. We use the PC-based FBNs constructed by our framework to distinguish the ASDs from NCs, and achieve 81.52% classification accuracy, which outperforms the baseline and state-of-the-art methods.

The remainder of this paper is organized as follows. In Section Materials and Methods, we introduce the material and methods. In particular, we first introduce the participants and review two related methods, i.e., PC and SR. Then, we reformulate PC into an optimization model and propose two specific PC-based FBN estimation methods, including the motivations, models, and algorithms for these two methods. In Section Results, we evaluate the two proposed methods with experiments on identifying ASD. In Section Discussion, we discuss our findings and prospects of our work. In Section Conclusion, we conclude the entire paper briefly.

## Materials and methods

### Data acquisition

In this paper, we have the same data set as the one in a recent study (Wee et al., 2016). Specifically, the data set includes resting-state fMRI (R-fMRI) data of 45 ASD subjects and 47 NC subjects (with ages between 7 and 15 years old). All these data are publicly available in the ABIDE database (Di et al., 2014). The demographic information of these subjects is summarized in Table 1. The ASD diagnostic was based on the autism criteria part in Diagnostic and Statistical Manual of Mental Disorders, 4th Edition, Text Revision (DSM-IV-TR). The psychopathology for differential diagnosis and comorbidities with Axis-I disorders was assessed by parent interview or participant interview. In particular, the parent interview was based on the Schedule of Affective Disorders and Schizophrenia for Children-Present and Lifetime Version (KSADS-PL) for children (<17.9 years old); the participant interview was based on the Structured Clinical Interview for DSM-IV-TR Axis-I Disorders, Non-patient Edition (SCID-I/NP) and the Adult ADHD Clinical Diagnostic Scale (ACDS) for adults (>18.0 years old). Exclusion of the comorbid ADHD needs to meet all criteria for ADHD (except for criterion E) in the DSM-IV-TR. Inclusion as a NC needs to exclude the entire current Axis-I disorders by KSADS-PL, SCID-I/NP, and ACDS interviews.

**Table 1:** Demographic information of the subjects.

	**ASD(N = 45)**	**NC(N = 47)**	***p*-values**
*Gender* (*M*/*F*)	36/9	36/11	0.2135^*^
*Age* (*year*±*SD*)	11.1 ± 2.3	11.0 ± 2.3	0.7773^†^
*FIQ* (*mean*±*SD*)	106.8 ± 17.4	13.3 ± 14.1	0.0510
*ADI*−*R* (*mean*±*SD*)	32.2 ± 14.3^‡^	–	–
*ADOS* (*mean*±*SD*)	13.7 ± 5.0	–	–

*ASD, Autism Spectrum Disorders; NC, Normal Control; FIQ, Full Intelligence Quotient; ADI-R, Autism Diagnostic Interview-Revised; ADOS, Autism Diagnostic Observation Schedule*.

* *The P-value was obtained by chi-squared test*.

† *The P-value was obtained by two-sample two-tailed t-test*.

‡ *Two patients do not have the ADI-R score*.

### Data preprocessing

All R-fMRI images were acquired using a standard echo-planar imaging sequence on a clinical routine 3T Siemens Allegra scanner. During 6 min R-fMRI scanning procedure, the subjects were required to relax with their eyes focusing on a white fixation cross in the middle of the black background screen projected on a screen. The imaging parameters include the flip angle = 90°, 33 slices, $TR/TE\;=\frac{2000}{15}ms$ with 180 volumes, and 4.0 *mm* voxel thickness. Data preprocessing was made by the standard software, statistical parametric mapping (SPM8 http://www.fil.ion.ucl.ac.uk/spm/software/spm8/). Specifically, the first 10 R-fMRI images of each subject were discarded to avoid signal shaking. The remainder images were calibrated as follows: (1) normalization to MNI space with resolution 3 × 3 × 3 *mm*^3^; (2) regression of nuisance signals (ventricle, white matter, global signals, and head-motion) with Friston 24-parameter model (Friston et al., 1996); (3) band-pass filtering (0.01–0.08 Hz); (4) signal de-trending. After that, the pre-processed BOLD time series signals were partitioned into 116 ROIs, according to the automated anatomical labeling (AAL) atlas (Tzourio-Mazoyer et al., 2002). At last, we put these time series into a data matrix *X* ∈ *R*^170×116^.

### Functional brain network estimation

After extracting the data matrix *X* from the R-fMRI data, we construct FBNs for these subjects based on the methods that will be given in the following subsections.

#### Related methods

It is well known that PC is possibly the most popular method to estimate FBNs (Smith et al., 2013). The mathematical expression of PC is defined as follows:

(1) $$W_{ij}=\frac{{\left(x_{i}-x_{i}\right)}^{T}\left(x_{j}-x_{j}\right)}{\sqrt{{\left(x_{i}-x_{i}\right)}^{T}\left(x_{i}-x_{i}\right)}\sqrt{{\left(x_{j}-x_{j}\right)}^{T}\left(x_{j}-x_{j}\right)}},$$

where $x_{i}\in R^{t}$ is the observed time course associated with ith brain regions, *t* is the number of time nodes, *x_i_* ∈ *R^t^* has all entries being the mean of the elements in *x*_*i*_, *i* = 1, 2, …, *n*, and *n* is the number of ROIs. Consequently, *x_i_* − *x_i_* is a centralized counterpart of *x*_*i*_.

As discussed previously, PC always generates dense FBNs. Thus, a threshold is often used to sparsify the PC-based FBNs (namely PC_threshold_), which can be expressed as follows:

(2) $$W_{ij}{}^{\left(new\right)}=\{\begin{array}{ll} W_{ij}, & W_{ij}>threshold\\ 0, & \;otherwise \end{array}$$

where ${W_{ij}}^{\left(new\right)}$ denotes the connection value between nodes i and j after thresholding.

Different from PC that measures the full correlation, SR is one of the widely-used schemes for modeling the partial correlation (Lee et al., 2011). The model of SR is shown as follows:

(3) $$min_{W}\sum_{i\;=\;1}^{n}{\left\|x_{i}-\sum_{j\;\neq \;i}W_{ij}x_{j}\right\|}^{2}+\lambda \sum_{j\;\neq \;i}\left|W_{ij}\right|,$$

or equivalently, its matrix form is

(4) $$\begin{array}{l} min_{W}{\left\|X-XW\right\|}_{F}^{2}+\lambda {\left\|W\right\|}_{1}\\ \text{ s}.\text{t}.W_{ii}=0,\forall i=1,\ldots ,n, \end{array}$$

where ${X=\left[x_{1},x_{2},\ldots \;,x_{n}\right]\in R}^{t\times n}$ represents the fMRI data matrix associated with a certain subject. Each column of *X* corresponds to the time course from a certain brain region. Note that the L_1_-norm regularizer in Equation (4) plays a key role in achieving a sparse and stable solution (Lee et al., 2011).

#### Our methods

As two typical examples, PC and SR have been demonstrated to be more sensitive than some complex higher-order methods (Smith et al., 2011). Therefore, in this paper, we mainly focus on these two methods, and we empirically found that PC tends to work better than SR in our experiments. However, compared with SR that controls the sparsity in an elegant mathematical model, the PC sparsifies the networks using an empirical hard threshold. Thus, a natural goal is to develop a new FBN estimation method that can inherit the robustness of PC and meanwhile has a flexible sparsification strategy as in SR. To this end, we first formulate the PC scheme as an optimization model, and then introduce an L_1_-norm regularizer into the model for achieving a sparse solution.

Without loss of generality, we suppose that the observed fMRI time series *x*_*i*_ of each node is centralized by *x_i_* − *x_i_* and normalized by $\sqrt{{\left(x_{i}-x_{i}\right)}^{T}\left(x_{i}-x_{i}\right)}$. That is, we define the new time series $x_{i}≜\frac{\left(x_{i}-x_{i}\right)}{\sqrt{{\left(x_{i}-x_{i}\right)}^{T}\left(x_{i}-x_{i}\right)}}$. As a result, we can simplify the PC as the formula $W_{ij}={x_{i}}^{T}x_{j}$, which can be easily proved to be the optimal solution of the following optimization problem:

(5) $$min_{W_{ij}}\sum_{i,j\;=\;1}^{n}{\left\|x_{i}-w_{ij}x_{j}\right\|}^{2}.$$

In fact, we first expand the objective function in Equation (5) as follows:

(6) $$\begin{array}{l} \sum_{i\;=\;1,j\;=\;1}^{n}{\left\|x_{i}-W_{ij}x_{j}\right\|}^{2}=\sum_{i\;=\;1,j=\;1}^{n}{\left(x_{i}-W_{ij}x_{j}\right)}^{T}\left(x_{i}-W_{ij}x_{j}\right)\\ \;=\sum_{i\;=1,j=\;1}^{n}\left(x_{i}{}^{T}x_{i}-2W_{ij}x_{i}x_{j}+W_{ij}{}^{2}x_{j}{}^{T}x_{j}\right)\\ \;=\sum_{i\;=\;1,j\;=\;1}^{n}\left(1-2W_{ij}x_{i}x_{j}+W_{ij}{}^{2}\right). \end{array}$$

Then, letting the derivative $\frac{d\overset{n}{\underset{i\;=\;1,j\;=\;1}{\sum }}\left(1-2W_{ij}x_{i}x_{j}+{W_{ij}}^{2}\right)}{dW_{ij}}$ be 0, we have the following result:

(7) $$2W_{ij}-2x_{i}x_{j}=0\to W_{ij}=x_{i}x_{j}.$$

Based on Equation (7), Equation (5) can be further formulated to a matrix form as follows:

(8) $$\underset{{}_{W}}{\min}{\left\|W-X^{T}X\right\|}_{F}^{2}.$$

Below, we will note that such an optimization view of PC can help improve the traditional PC and further develop new flexible FBN estimation methods.

Motivated by the model of SR, we can naturally incorporate a regularized term into the objective function of Equation (8) for constructing a new platform to estimate FBNs. More specifically, the platform can be formulated using a matrix-regularized learning framework as follows:

(9) $$\begin{array}{l} \underset{W}{\min}{\left\|W-X^{T}X\right\|}_{F}^{2}+\lambda R\left(W\right)\\ \text{ S}.\text{t}.\text{W}\in \Delta , \end{array}$$

where *R*(*W*) is a regularized term, λ is a trade-off parameter, and is a set of additional constraints on the constructed FBNs, such as, the positive definiteness and non-negativity, etc.

Here, we argue that the PC-based FBN learning framework shown in Equation (9) has two advantages: (1) it is statistically robust and scales well, without the ill-posed problem involved in the SR-based (partial correlation) method; (2) biological/physical priors (e.g., sparsity) can be naturally introduced into the model in the form of regularizer for constructing more meaningful FBNs. In order to illustrate the flexibility of the proposed framework, we develop two specific remodeling PC-based FBN estimation methods (I and II) that will be discussed below, respectively.

### Method I: remodeling PC-based FBN with a sparsity prior

As pointed out previously, the hard-threshold scheme is an effective scheme to sparsify the FBNs, which can be regarded as a special format of the L_1_-norm. However, generally, the threshold selection is empirical without an elegant mathematical representation. In addition, it is hard to incorporate other biological/physical priors into FBN construction task. In this paper, based on the proposed FBN learning framework, we first introduce the L_1_-norm as an instantiation of the regularized term *R*(*W*), resulting in a new remodeling PC-based FBN estimation model (namely PC_sparsity_) as follows:

(10) $$min_{W}\;{\left\|W-X^{T}X\right\|}_{F}^{2}+\lambda {\left\|W\right\|}_{1},$$

where λ is a regularized parameter for controlling the sparsity of *W*. Obviously, the PC_sparsity_ reduces to the original PC when λ = 0. Besides the L_1_-norm, there are some alternative regularizers, such as, the log-sum strategy (Shen et al., 2013), can be introduced in the proposed framework to sparsify FBNs. Here, we select the L_1_-norm since it is simple and popular.

The objective function of Equation (8) is convex but indifferentiable due to the L_1_-norm regularizer. A number of algorithms have been developed to address the indifferentiable convex optimization problem in the past few years (Donoho and Elad, 2003; Meinshausen and Bühlmann, 2006; Tomioka and Sugiyama, 2009; Zhao, 2013). Here, we employ the proximal method (Combettes and Pesquet, 2011; Bertsekas, 2015) to solve Equation (10) for the main reason of its simplicity and efficiency. In particular, we first consider the fidelity term $f\left(X,W\right)=||W-X^{T}X|{|}_{F}^{2}$ in Equation (10), which is differentiable, and its gradient is $\nabla_{W}f\left(X,W\right)=2\left(W-X^{T}X\right)$. As a result, it is easy to get the following gradient descent step:

(11) $$W_{k}=W_{k-1}-\alpha_{k}\nabla f\left(X,W_{k-1}\right),$$

where α_*k*_ denotes the step size of the gradient descent.

Then, according to Combettes and Pesquet (2011) and the definition Data Acquisition therein, the proximal operator of L_1_-norm regularizer on *W* can be given as the following soft-threshold operation:

(12) $$proximal_{\lambda {\left\|\cdot \right\|}_{1}}\left(W\right)={\left[sgn\left(W_{ij}\right)\times \max\left(abs\left(W_{ij}\right)-\lambda \right),0\right]}_{p\times p}.$$

Finally, we use the proximal operation *proximal*_λ||•||_1__ in Equation (12) on *W* to keep *W* in the “feasible region” (regularized by the L_1_-norm) after each gradient descent step. Consequently, we get a simple algorithm for solving Equation (10) as shown in Table 2.

**Table 2:** Algorithm of PC-based FBN estimation with a sparse prior.

**Input**: *X* //observed data
**Output**: *W* //functional brain network
**Initialize** *W*;
**while** *not converge*
*W* ← *W* − α (*W* − *X*^*T*^*X*)*;*
*W* ← *proximal*_λ||•||_1__(*W*) = [*sgn*(*W*_*i, j*_) × *max*(*abs*(*W*_*i, j*_) − λ, 0)]_*p* × *p*_;
**end**

### Method II: remodeling PC-based FBN with a scale-free prior

It is well known that a brain network has more topological structures than just sparsity (Sporns, 2011) such as, modularity (Qiao et al., 2016), hierarchy (Zhou et al., 2006), small-worldness (Watts and Strogatz, 1998; Achard et al., 2006), clustering (White et al., 1986), degeneracy (Tononi et al., 1999), and scale-free (Eguíluz et al., 2005; Li et al., 2005). In order to verify the flexibility of the proposed framework in Equation (9), we develop a new PC-based FBN estimation model by incorporating a scale-free prior. Consequently, we have the following optimization model (namely PC_scale−free_):

(13) $$min_{W}{\left\|W-X^{T}X\right\|}_{F}^{2}+\lambda \sum_{i,j\;=\;1}^{n}\gamma_{ij}\left|W_{ij}\right|.$$

Similar to the PC_sparsity_ in Equation (10), λ is the regularized parameter. In order to incorporate the node degree information, a weight γ_*ij*_ related to the node degree of each *W*_*ij*_ is introduced in the PC_scale−free_ model, which essentially makes the PC_scale−free_ be a weighted version of PC_sparsity_. We argue that such a weighted extension can achieve a scale-free network by assigning the weight γ_*ij*_ properly as discussed below.

Note that the fidelity term $f\left(X,W\right)=||W-X^{T}X|{|}_{F}^{2}$ of Equation (13) is the same as the one in Equation (10). Thus, the two problems share the same gradient descent step as shown in Equation (11). Then, we consider the regularized term $\lambda \overset{n}{\underset{i,j\;=\;1}{\sum }}\gamma_{ij}|W_{ij}|$. Based on the definition of the proximal operation, we can easily get the proximal operator of the weighted L_1_-norm regularizer on *W* as follows:

(14) $$\begin{array}{l} proximal_{\lambda \gamma_{ij}{\left\|\cdot \right\|}_{1}}\left(W\right)=[sgn\left(W_{i,j}\right)\times \max(abs\left(W_{ij}\right)\\ \;-\lambda \times \gamma_{ij}),0{]}_{p\times p}, \end{array}$$

which is exactly a weighted version of the soft threshold operation. Since the node degree of the brain network tends to follow the power law distribution (Barabási and Bonabeau, 2003; Eguíluz et al., 2005; Cecchi et al., 2007; Lin and Ihler, 2011), we assume that the hub nodes cover more useful connections (closely related to the neural disorders), while the non-hub nodes cover weak or noisy connections. Therefore, compared with the PC_sparsity_ method that equally treats each edge (or link) of the FBN, the PC_scale−free_ method penalizes more on the nodes with small degree, and penalizes less on the nodes with large de gree. According to Equation (14), a big γ_*ij*_ may increase the possibility that *W*_*ij*_ shrinks to zero, which in turn tend to result in a sparse vector *W*_*i*_ = (*W*_*i*1_, *W*_*i*2_, …, *W*_*ip*_), and then a small degree of node *i*. Conversely, a small γ_*ij*_ may result in a big degree of node *i*. In other words, the parameter γ_*ij*_ should have an inverse relationship with the node degree (Peng et al., 2009; Lin and Ihler, 2011). Thus, we assume that γ_*ij*_ has the following form:

(15) $$\gamma_{ij}=e^{-(\frac{1}{\sum_{z\;=\;1}^{n}\left|w_{iz}\right|+\varepsilon }+\frac{1}{\sum_{z\;=\;1}^{n}\left|w_{jz}\right|+\varepsilon })},$$

where ε is a small number for preventing the denominator in Equation (15) to be zero. In our experiment, we simply set ε = 0.0001. As a consequence, we get the following alternating optimization algorithm. In each iteration, with a fixed *W*, the parameter γ_*ij*_ can be easily calculated by Equation (15), and then by fixing the value of each parameter γ_*ij*_, we update *W* by solving Equation (13). We summarize the algorithm for solving Equation (13) in the following Table 3.

**Table 3:** Algorithm of PC-based FBN estimation with a scale-free prior.

**Input**: *X* //observed data
**Output**:*W* //functional brain network
**Initialize** γ_*ij*_ = 1;
**while** *not converge*
$W\leftarrow argmin_{W}||W-X^{T}X|{|}_{F}^{2}+\lambda \overset{n}{\underset{i,j=1}{\sum }}\gamma_{ij}|W_{ij}|$*;* //by a weighted version of Algorithm in Table 2.
$\gamma_{ij}\leftarrow e^{-\left(\frac{1}{\overset{n}{\underset{z=1}{\sum }}|w_{iz}|+\varepsilon }+\frac{1}{\overset{n}{\underset{z=1}{\sum }}|w_{jz}|+\varepsilon }\right)}$;
**end**

#### Experimental setting

After obtaining the FBNs of all subjects, the main task comes to use the constructed FBNs to train a classifier for identifying ASDs from NCs. Since the FBN matrix is symmetric, we just use its upper triangular elements as input features for classification. Even so, the dimensions of the features are still too high to train a classifier with good generalization, due to the limited training samples in this study. Therefore, we first conduct a feature filtering operation before training the classification. Specifically, the classification pipeline includes the following two main steps.

**Figure 1:**
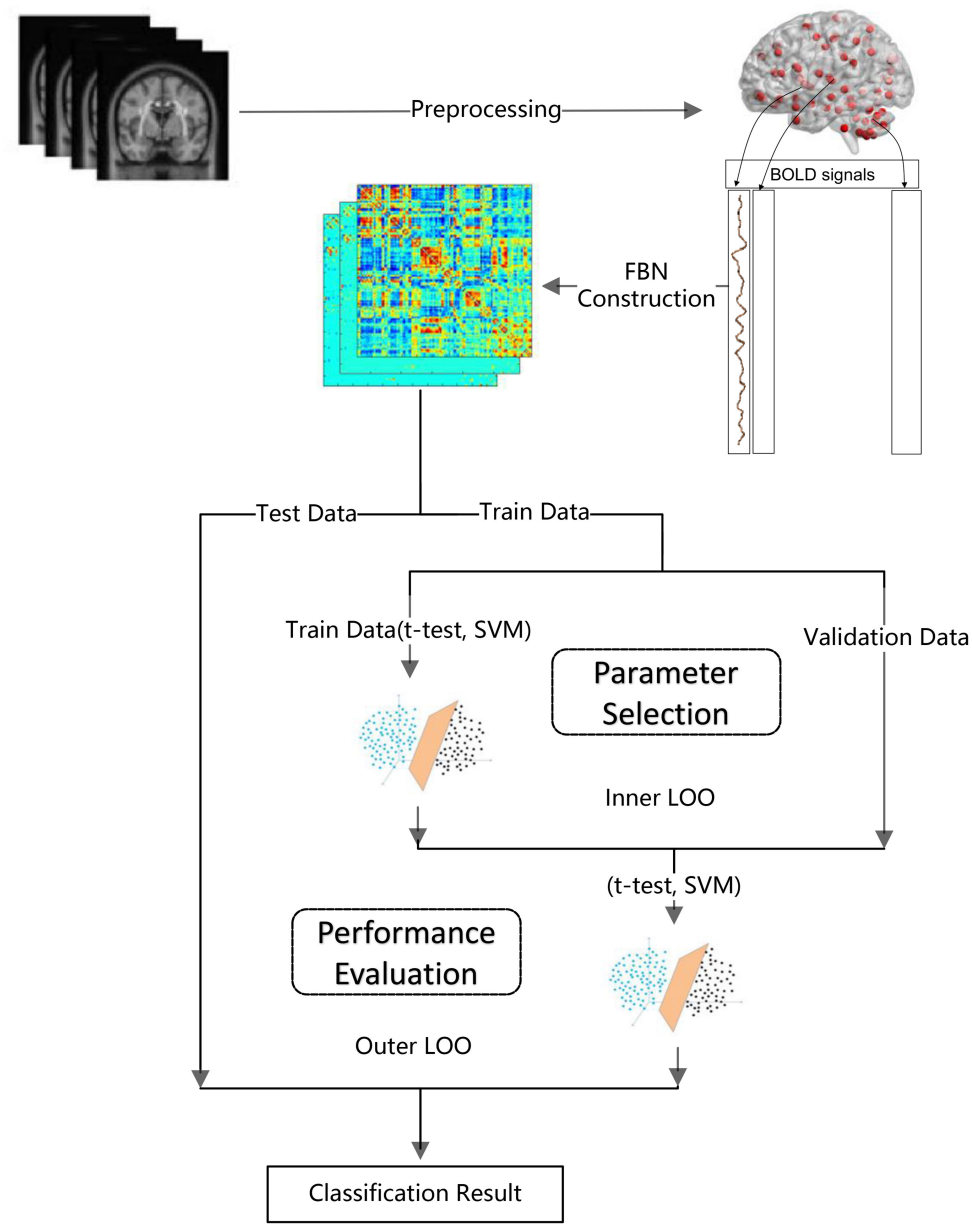
The main procedure of FBN-based classification used in our experiment.

> ***Step 1****: FBN construction based on PC*_threshold_^2^, *SR, PC*_sparsity_, *and PC*_scale−free_*, respectively*. Note that each FBN construction method involves a free parameter, e.g., the threshold parameter in PC_threshold_ and the regularized parameter in the other methods. Therefore, in this step, we construct multiple FBNs based on different parametric values, and then select the optimal FBN (for each method) based on a separate parameter selection procedure, as shown in Figure 1.
> ***Step 2:*** *Feature selection and classification using t-test (with p < 0.05) and linear SVM (with default parameter C* = *1), respectively*. As pointed out in Wee et al. (2014), both the feature selection and classifier design have a big influence on the final accuracy. However, in this paper, we only adopt the simplest feature selection method and the most popular used SVM classifier (Chang and Lin, 2007), since our main focus is FBN estimation. In other words, it would be difficult to conclude whether the FBN construction methods or the feature selection/classification methods contribute to the ultimate performance.

The detailed experimental procedure (including a subprocedure for parameter selection) is shown in Figure 1. Due to the small sample size, we use the leave one out (LOO) cross validation strategy to verify the performance of the methods, in which only one subject is left out for testing while the others are used to train the models and get the optimal parameters. For the choice of the optimal parameters, an inner LOO cross-validation is further conducted on the training data by grid-search strategy. More specifically, for the regularized parameter λ, the candidate values range in [0.05, 0.1, …, 0.95, 1]; for the hard threshold of PC_threshold_, we use 20 sparsity levels ranging in [5, 10, …, 95, 100]. For example, the 90% means that 10% of the weak edges are filtered out from the FBN.

## Results

### Network visualization

**Figure 2:**
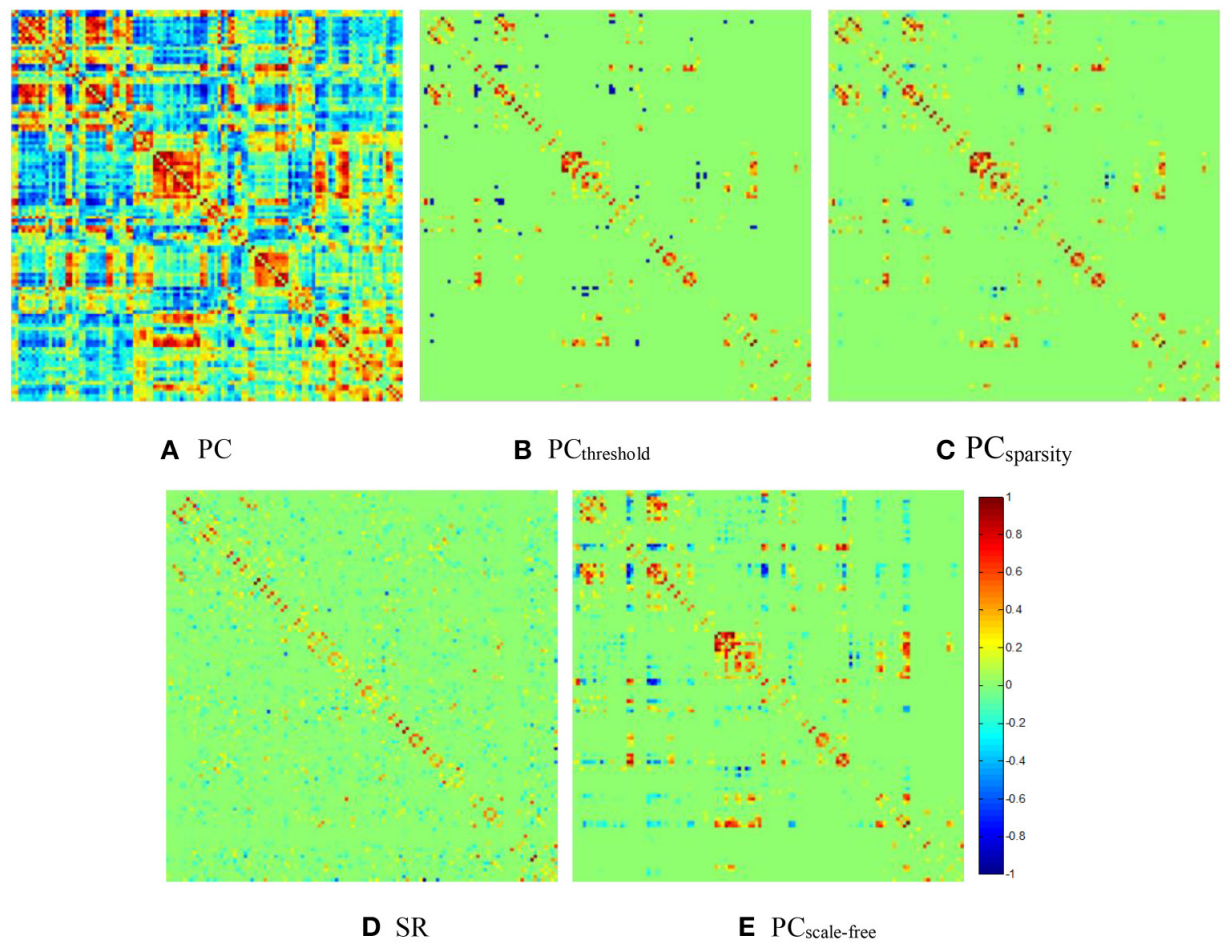
The FBN adjacency matrices of a certain subject, constructed by different methods. **(A)** PC, **(B)** PC_threshold_, **(C)** PC_sparsity_, **(D)** SR, and **(E)** PC_scale−free_.

For visual comparison of the FBN constructed by PC_threshold_, SR, PC_sparsity_ and PC_scale−free_ methods, we first show the FBN adjacency matrices^3^ *W* constructed by different methods in Figure 2.

It can be observed from Figure 2 that both (Figure 2B) PC_threshold_ and (Figure 2C) PC_sparsity_ can remove the noisy or weak connections from the dense FBN constructed directly by the original PC. Moreover, the topology of the FBN estimated by PC_sparsity_ is similar to that of PC_threshold_, because (1) both methods employ the same data-fidelity term, and (2) the sparsification strategy behind PC_sparsity_ (i.e., the soft-thresholding scheme) is based on the result of PC_threshold_ (i.e., the hard-thresholding scheme). In contrast, the FBN constructed by SR has a topology highly different from those of PC_threshold_ and PC_sparsity_, since it uses a different data-fidelity term [i.e., the first term in Equation (4)]. More interestingly, compared with PC_threshold_ and PC_sparsity_, the FBN estimated by (Figure 2E) PC_scale−free_ has a clearer hub structure, due to the use of a weighted L_1_-norm regularizer.

**Figure 3:**
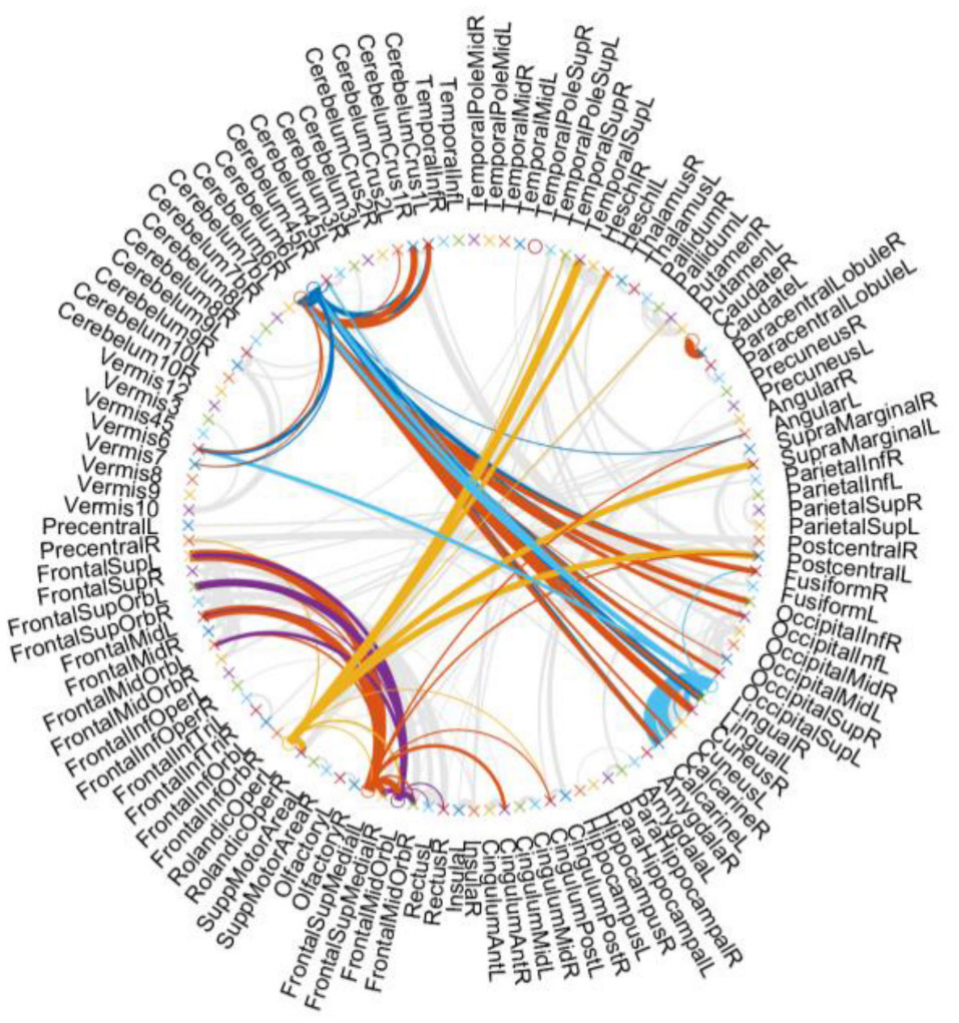
Brain connections estimated by PC_scale−free_.

For showing the hub structure more clearly, we plot the brain connections estimated by PC_scale−free_ in Figure 3, where the width of each arc represents the weight of the connection between two endpoints. Furthermore, we color the connections from the hub nodes, while showing other connections in gray for better visualization. In Figure 3, it can be interestingly observed that (1) the hub nodes are only a small proportion of the whole brain regions, illustrating the scale-free characteristic of the constructed FBN; (2) the hub nodes mainly locate at the brain regions, including the Cerebellum, Frontal, Rolandic, and Lingual, etc.

**Figure 4:**
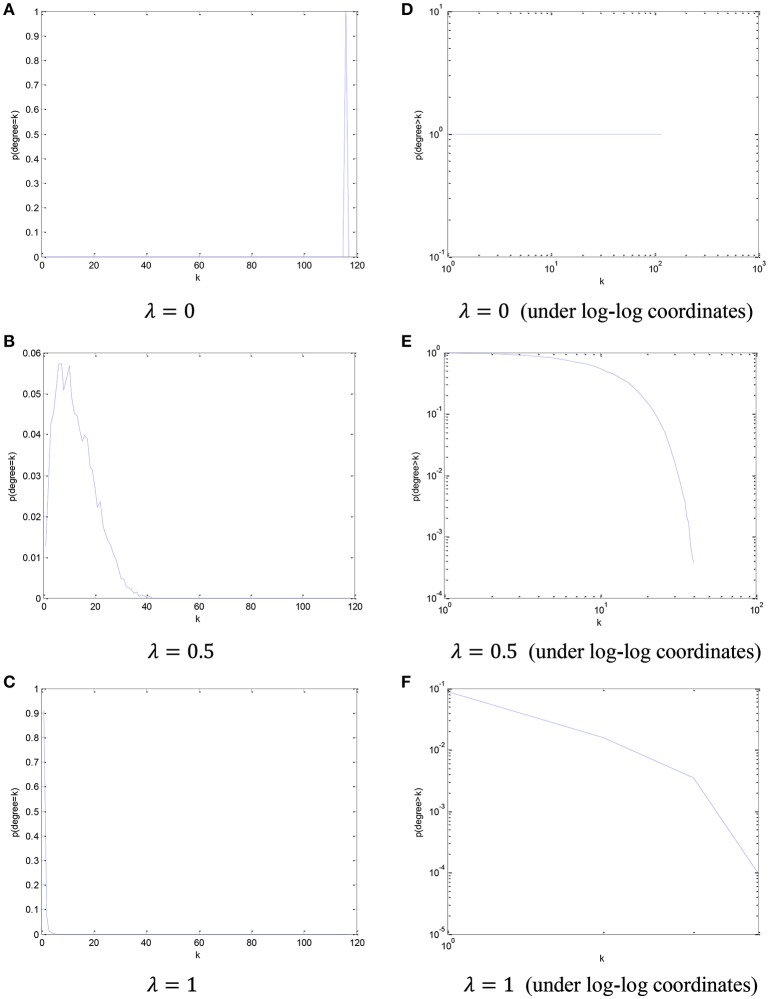
The distribution of node degree and the corresponding cCDF under log-log coordinates with respect to different parameter λ. **(A)** λ = 0, **(B)** λ = 0.5, **(C)** λ = 1, **(D)** λ = 0 (under log-log coordinates), **(E)** λ = 0.5 (under log-log coordinates), and **(F)** λ = 1 (under log-log coordinates).

In order to visualize the relationship between the parameter λ in the PC_scale−free_ model and the node degree, we simply count the number of the nodes from all participants in this dataset based on different node degree, and plot its cCDF (complementary cumulative distribution function) under log-log coordinates. The distribution of node degree results based on different values of parameter λ are shown in Figure 4.

Based on the results in Figure 4, we can find that, with the increase of the parametric value, the node degree distribution tends to be more scale-free.

For verifying the effectiveness of the regularizer and quantifying the scale-free topology of FBN constructed by PC_scale−free_ and PC_sparsity_, we employ the s-metric (Li et al., 2005) to compute the corresponding scale-free measures.

(16) $$S(W)=\sum d_{i}d_{j},$$

where *d*_*i*_ means the degree of the node i, and *S*(*W*) is the value of the s-metric for the network W. Since the s-metric relies on the number of the connects in FBN, and the network threshold affects the degree and scale-free measures significantly for these two methods. In this paper, as an example, we construct the FBN by PC_scale−free_ (λ = 0.5), and then find the FBN constructed by PC_sparsity_ with the same number of connects. Based on Equation (14), the s-metric of the FBN constructed by PC_sparsity_ is 18313091, and the one by PC_scale−free_ is 27862470. We note that the PC_scale−free_ has a higher s-metric value than PC_sparsity_. Since the high s-metric value is achieved by connecting high degree nodes to each other, the FBN constructed by PC_scale−free_ can obtain more hub-nodes than the FBN constructed by PC_sparse_. Thus, the brain network constructed by PC_scale−free_ tends to be more “scale-free”.

### ASD identification

The ASD vs. NC classification results on ABIDE dataset are given in Table 4. The remodeling method (PC_scale−free_) achieves the best accuracy in this experiment. In addition, the results of Wee et al.'s method available from Wee et al. (2016) are also provided in Table 4 as a reference.

**Table 4:** Classification performance corresponding to different FBN estimation methods on ABIDE dataset.

**Method**	**Accuracy**	**Sensitivity**	**Specificity**
PC_threshold_	63.04	62.22	63.83
SR	55.43	60.00	51.06
(Wee et al., 2016)	70.70	81.40	61.20
PC_sparsity_	64.13	68.89	59.57
PC_scale−free_	**81.52**	**84.44**	**78.72**

A set of quantitative measurements, including accuracy, sensitivity, and specificity, are used to evaluate the classification performance of four different methods (PC_threshold_, SR, PC_sparsity_ and PC_scale−free_). The mathematical definition of these three measures are given as follows:

(17) $$Accuracy=\frac{TruePostive+TrueNegative}{\begin{array}{c} TruePostive+FalsePostive\\ +TrueNegative+FalseNegative \end{array}},$$

(18) $$Sensitivity=\frac{TruePostive}{TruePostive+FalseNegative},$$

(19) $$Specificity=\frac{TrueNegative}{TrueNegative+FalsePostive},$$

Here, *TruePositive* is the number of the positive subjects that are correctly classified in the ASD identification task. Similarly, *TrueNegative, FalsePostive*, and *FalseNegative* are the numbers of their corresponding subjects, respectively.

#### Sensitivity to network model parameters

**Figure 5:**
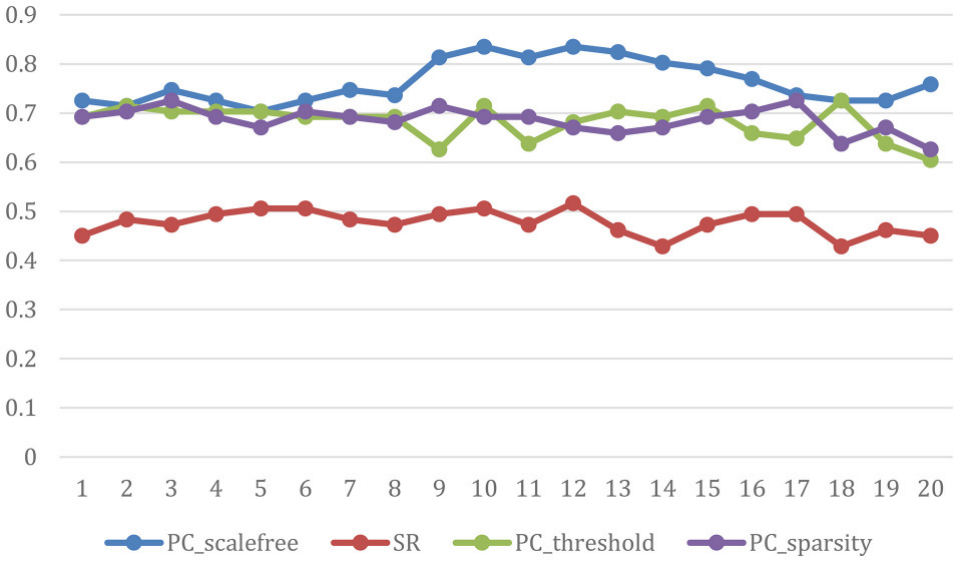
Classification accuracy based on the networks estimated by four different methods of 20 regularized parameters. The results are obtained by LOO test on all subjects in ABIDE.

The ultimate classification accuracy is particularly sensitive to the network model parameters. In Figure 5, we show the classification accuracy corresponding to different parametric combination (i.e., [0.05, 0.1, …, 0.95, 1] for SR, PC_sparsity_, and PC_scale−free_ [5%, 10%, …, 95%, 100%] for PC_threshold_) in 4 different methods. In addition, the classification accuracy is computed by the LOO test on all of the subjects.

## Discussion

The FBN commonly has more “structures” than just sparsity (Smith et al., 2011; Sporns, 2011). In this work, we remodel the PC-based method into an optimization model for incorporating some of these structures such as, scale-free property. The proposed models were verified on the ABIDE dataset for ASD vs. NC classification. Based on the results, we give the following brief discussion.

1. The accuracy of the PC-based methods outperforms the SR method on our used dataset. A possible reason is that the SR implicitly involves an inverse operation on the covariance matrix, which tends to be ill-posed due to the limited sample size and high-dimensional features. In fact, a recent study (Qiao et al., 2016) also notes a similar problem that the performance of SR-based method drops significantly with the increase of the feature dimension. In contrast, the PC-based methods can be derived directly from the covariance matrix without the inverse operation, and thus works robustly and also generally scales well.
2. The performance of PC_sparsity_ in our experiments is similar to that of the hard-threshold counterpart PC_threshold_, because both methods share the same data-fidelity term and a similar sparsification scheme (i.e., hard threshold for PC_threshold_ while soft threshold for PC_sparsity_). The subtle difference of the results between these two methods may be due to the regularized parameters (e.g., hard threshold in PC and λ in PC_sparsity_). However, we argue that the model of PC_sparsity_ is more flexible than PC_threshold_. For example, it can be naturally extended to a weighted version, namely PC_scale−free_, for better performance.
3. The proposed PC_scale−free_ method achieves the best classification accuracy among all the methods. In our opinion, this is mainly due to its power for modeling the hub node in a network that may cover the useful connections closely related to neural disorders. Interestingly, it outperforms Wee et al.'s method (Wee et al., 2016), which used the same NYU dataset, even though the latter employs more sophisticated feature selection and classification strategy. In addition, the proposed PC_scale−free_ method provides an empirical evidence that a suitable biological/physical prior can be used to guide the estimation of better FBNs.

In addition, we further conduct experiments for verifying the effectiveness of the proposed methods on a non-ASD fMRI dataset from ADNI, and find that the PC_scale−free_ methods still achieve the best accuracy. Since the main focus of this paper is on ASD identification, we supply the details of the dataset and experimental results in a Supplementary Material. The results show that the proposed method tends to generalize well on both ASD and non-ASD datasets. In other words, the idea for estimating FBN in this paper is general and independent of the used datasets. However, there are several limitations in the proposed methods that need to be improved in the future work.

1. We use the L_1_-norm (or weighed L_1_-norm) as a regularizer to estimate sparse (or scale-free) FBNs for the subjects one by one. However, the FBNs of different subjects tend to share some similar structures (Wee et al., 2014; Yu et al., 2016) and thus the proposed method may lose such group information. Therefore, in the future work, we need to adopt the development and application of “group constraint” such as, Group LASSO (Yuan and Lin, 2006) for addressing this problem.
2. In this paper, the ratio of male to female participants is substantially 5 to 1. According to a recent finding (Lai et al., 2013), the gender is one of the obvious sources of heterogeneity in ASD. Therefore, in the future work, we need to consider this issue for reducing the effect of heterogeneity.

## Conclusion

Pearson's correlation is the most commonly used scheme in estimating FBNs due to its simplicity, efficiency and robustness. However, the PC scheme is inflexible due to the difficulty of incorporating informative priors. In this paper, we remodel the PC into an optimization framework, based on which the biological priors or assumptions can be naturally introduced in the form of regularizers. More specifically, based on this framework, we propose two PC-based FBN estimation methods, namely PC_sparsity_ and PC_scale−free_, which can effectively encode sparse and scale-free priors, respectively. Finally, we use these constructed FBNs to classify the ASDs from NCs, and get an 81.52% accuracy, which outperforms the baseline and state-of-the-art methods. On the other hand, the topology of FBN is much more than just the sparsity and scale-free. Therefore, it is a potentially valuable topic to incorporate other biological/physical priors in constructing FBNs.

## Author contributions

All authors developed remodeling algorithm, architecture. WL, ZW, LZ, and LQ designed the evaluation experiments. DS preprocessed the fMRI. All authors contributed to preparation of the article, figures, and charts.

### Conflict of interest statement

The authors declare that the research was conducted in the absence of any commercial or financial relationships that could be construed as a potential conflict of interest.

## Footnotes

^1^In this paper, we will interchangeably use regions of interest (ROIs) and brain regions to denote network/graph nodes for the convenience of presentation.

^2^In order to improve the flexibility of PC and conduct fair comparison, we introduce a hard-thresholding parameter in PC by reducing a proportion of weak connections.

^3^The adjacency matrix is an algebraic expression of a graph (or network). The elements of the matrix indicate the connection strength of the node pairs in the graph. Here, for the convenience of comparison among different methods, all the weights are normalized to the interval [-1 1].

## References

[B1] Achard S. Salvador R. Whitcher B. Suckling J. Bullmore E. (2006). A resilient, low-frequency, small-world human brain functional network with highly connected association cortical hubs. J. Neurosci. 26, 63–72. 10.1523/JNEUROSCI.3874-05.2006 16399673 PMC6674299

[B2] Allen G. Courchesne E. (2003). Differential effects of developmental cerebellar abnormality on cognitive and motor functions in the cerebellum: an fMRI study of autism. Am. J. Psychiatry 160, 262–273. 10.1176/appi.ajp.160.2.262 12562572

[B3] Anderson J. S. Druzgal T. J. Froehlich A. Dubray M. B. Lange N. Alexander A. L.. (2011). Decreased interhemispheric functional connectivity in autism. Cereb. Cortex 21, 1134–1146. 10.1093/cercor/bhq190 20943668 PMC3077433

[B4] Baio J. (2014). Prevalence of autism spectrum disorder among children aged 8 years-autism and developmental disabilities monitoring network, 11 sites, United States, 2010. MMWR 63, 1–21. 24670961

[B5] Barabási A. L. Bonabeau E. (2003). Scale-free networks. Sci. Am. 288, 60–69. 10.1038/scientificamerican0503-60 12701331

[B6] Bertsekas D. P. (2015). Incremental gradient, subgradient, and proximal methods for convex optimization: a survey. Optimization 2, 691–717.

[B7] Brambilla P. Hardan A. Nemi S. U. D. Perez J. Soares J. C. Barale F. (2003). Brain anatomy and development in autism: review of structural MRI studies. Brain Res. Bull. 61, 557–569. 10.1016/j.brainresbull.2003.06.001 14519452

[B8] Brunetti M. Belardinelli P. Gratta C. D. Pizzella V. Penna S. D. Ferretti A. (2006). Human brain activation elicited by the localization of sounds delivering at attended or unattended positions: an fMRI/MEG study. Cogn. Process. 7, 116–117. 10.1007/s10339-006-0093-3

[B9] Cecchi G. A. Rao A. R. Centeno M. V. Baliki M. Apkarian A. V. Chialvo D. R. (2007). Identifying directed links in large scale functional networks: application to brain fMRI. BMC Cell Biol. 8:S5. 10.1186/1471-2121-8-S1-S5 17634095 PMC1924510

[B10] Chang C. C. Lin C. J. (2007). LIBSVM: a library for support vectormachines. ACM Trans. Intell. Syst. Technol. 2, 389–396. 10.1145/1961189.1961199

[B11] Combettes P. L. Pesquet J. C. (2011). Proximal Splitting Methods in Signal Processing. Fixed-Point Algorithms for Inverse Problems in Science and Engineering. New York, NY: Springer 10.1007/978-1-4419-9569-8_10

[B12] Delmonte S. Gallagher L. O'Hanlon E. McGrath J. Balsters J. H. (2012). Functional and structural connectivity of frontostriatal circuitry in autism spectrum disorder. Front. Hum. Neurosci. 7:430. 10.3389/fnhum.2013.00430 23964221 PMC3734372

[B13] Di M. A. Yan C. G. Li Q. Denio E. Castellanos F. X. Alaerts K. (2014). The autism brain imaging data exchange: towards a large-scale evaluation of the intrinsic brain architecture in autism. Mol. Psychiatry 19, 659–667. 10.1038/mp.2013.78 23774715 PMC4162310

[B14] Donoho D. L. Elad M. (2003). Optimally sparse representation in general (nonorthogonal) dictionaries via l. Proc. Natl. Acad. Sci. U.S.A. 100, 2197–2202. 10.1073/pnas.0437847100 16576749 PMC153464

[B15] Ecker C. Rocha-Rego V. Johnston P. Mourao-Miranda J. Marquand A. Daly E. M.. (2010). Investigating the predictive value of whole-brain structural MR scans in autism: a pattern classification approach. Neuroimage 49, 44–56. 10.1016/j.neuroimage.2009.08.024 19683584

[B16] Eguíluz V. M. Chialvo D. R. Cecchi G. A. Baliki M. Apkarian A. V. (2005). Scale-free brain functional networks. Phys. Rev. Lett. 94:018102. 10.1103/PhysRevLett.94.018102 15698136

[B17] Fan Y. Browndyke J. N. (2010). MCI diagnosis via manifold based classification of functional brain networks. Alzheimers Dementia 6:S16 10.1016/j.jalz.2010.05.044

[B18] Fornito A. Zalesky A. Bullmore E. T. (2016). Fundamentals of brain network analysis. Sociol. Q. 47, 471–495.

[B19] Friston K. J. Williams S. Howard R. Frackowiak R. S. J. Turner R. (1996). Movement-Related effects in fMRI time-series. Magn. Reson. Med. 35, 346–355. 10.1002/mrm.1910350312 8699946

[B20] Frith U. Happé F. (2005). Autism spectrum disorder. Curr. Biol. 15, 786–790. 10.1016/j.cub.2005.09.033 16213805

[B21] Gillberg C. (1993). Autism and related behaviors. J. Intell. Disabil. Res. 37(Pt 4), 343–372. 10.1111/j.1365-2788.1993.tb00879.x 8400719

[B22] Gotts S. J. Simmons W. K. Milbury L. A. Wallace G. L. Cox R. W. Martin A. (2012). Fractionation of social brain circuits in autism spectrum disorders. Brain 135, 2711–2725. 10.1093/brain/aws160 22791801 PMC3437021

[B23] Horwitz B. (2003). The elusive concept of brain connectivity. Neuroimage 19:466. 10.1016/S1053-8119(03)00112-5 12814595

[B24] Huang S. Li J. Sun L. Liu J. Wu T. Chen K. (2009). Learning brain connectivity of Alzheimer's disease from neuroimaging data, in Advances in Neural Information Processing Systems 22: Conference on Neural Information Processing Systems 2009. Proceedings of A Meeting Held 7-10 December 2009 (Vancouver, BC), 808–816.

[B25] Jin H. L. Durand R. Gradinaru V. Feng Z. Goshen I. Kim D. S. (2010). Global and local fMRI signals driven by neurons defined optogenetically by type and wiring. Nature 465, 788–792. 10.1038/nature09108 20473285 PMC3177305

[B26] Kevin W. Doug W. Matthias S. Gerhard S. (2008). Correspondence of Visual Evoked Potentials with FMRI signals in human visual cortex. Brain Topogr. 21, 86–92. 10.1007/s10548-008-0069-y 18841455

[B27] Lai M. C. Lombardo M. V. Suckling J. Ruigrok A. N. Chakrabarti B. Ecker C.. (2013). Biological sex affects the neurobiology of autism. Brain 136, 2799–2815. 10.1093/brain/awt216 23935125 PMC3754459

[B28] Lee H. Lee D. S. Kang H. Kim B. N. Chung M. K. (2011). Sparse brain network recovery under compressed sensing. IEEE Trans. Med. Imaging 30, 1154–1165. 10.1109/TMI.2011.2140380 21478072

[B29] Li L. Alderson D. Doyle J. C. Willinger W. (2005). Towards a theory of scale-free graphs: definition, properties, and implications. Internet Math. 2, 431–523. 10.1080/15427951.2005.10129111

[B30] Liu F. Wee C. Y. Chen H. Shen D. (2012). Inter-modality relationship constrained multi-task feature selection for AD/MCI classification. Med. Image Comput. Comput. Assist. Interv. 16, 308–315. 24505680 10.1007/978-3-642-40811-3_39 PMC4109067

[B31] Lin Q. Ihler A. T. (2011). Learning scale free networks by reweighted L_1_ regularization. J. Mach. Learn. Res. 15, 40–48.

[B32] Lo Y. C. Soong W. T. Gau S. F. Wu Y. Y. Lai M. C. Yeh F. C.. (2011). The loss of asymmetry and reduced interhemispheric connectivity in adolescents with autism: a study using diffusion spectrum imaging tractography. Psychiatry Res. 192, 60–66. 10.1016/j.pscychresns.2010.09.008 21377337

[B33] Lord C. Cook E. H. Leventhal B. L. Amaral D. G. (2000). Autism spectrum disorders. Neuron 41, 541–543. 10.1016/S0896-6273(00)00115-X 11144346

[B34] Lord C. Jones R. M. (2012). Annual research review: re-thinking the classification of autism spectrum disorders. J. Child Psychol. Psychiatry Allied Discipl. 53, 490–509. 10.1111/j.1469-7610.2012.02547.x 22486486 PMC3446247

[B35] Mason M. F. Norton M. I. Horn J. D. V. Wegner D. M. Grafton S. T. Macrae C. N. (2007). Wandering minds: the default network and stimulus-independent thought. Science 315, 393–395. 10.1126/science.1131295 17234951 PMC1821121

[B36] Meinshausen N. Bühlmann P. (2006). High-dimensional graphs and variable selection with the Lasso. Ann. Stat. 34, 1436–1462. 10.1214/009053606000000281

[B37] Nielsen J. A. Zielinski B. A. Fletcher P. T. Alexander A. L. Lange N. Bigler E. D.. (2013). Multisite functional connectivity MRI classification of autism: ABIDE results. Front. Hum. Neurosci. 7:599. 10.3389/fnhum.2013.00599 24093016 PMC3782703

[B38] O'Roak B. J. Vives L. Fu W. Egertson J. D. Stanaway I. B. Phelps I. G.. (2012). Multiplex targeted sequencing identifies recurrently mutated genes in autism spectrum disorders. Science 338, 1619–1622. 10.1126/science.1227764 23160955 PMC3528801

[B39] Peng J. Wang P. Zhou N. Zhu J. (2009). Partial correlation estimation by joint sparse regression models. J. Am. Stat. Assoc. 104, 735–746. 10.1198/jasa.2009.0126 19881892 PMC2770199

[B40] Power J. D. Mitra A. Laumann T. O. Snyder A. Z. Schlaggar B. L. Petersen S. E. (2013). Methods to detect, characterize, and remove motion artifact in resting state fMRI. Neuroimage 84, 320–341. 10.1016/j.neuroimage.2013.08.048 23994314 PMC3849338

[B41] Qiao L. Zhang H. Kim M. Teng S. Zhang L. Shen D. (2016). Estimating functional brain networks by incorporating a modularity prior. Neuroimage 141, 399–407. 10.1016/j.neuroimage.2016.07.058 27485752 PMC5338311

[B42] Rosa M. J. Portugal L. Hahn T. Fallgatter A. J. Garrido M. I. Shawe-Taylor J.. (2015). Sparse network-based models for patient classification using fMRI. Neuroimage 105, 493–506. 10.1016/j.neuroimage.2014.11.021 25463459 PMC4275574

[B43] Segal D. L. (2013). Diagnostic and statistical manual of mental disorders (DSM-IV-TR), in Corsini Encyclopedia of Psychology, eds Weiner I. Craighead W. E. (Hoboken NJ: John Wiley & Sons, Inc.), 495–497.

[B44] Shen Y. Fang J. Li H. (2013). Exact reconstruction analysis of log-sum minimization for compressed sensing. IEEE Signal Process. Lett. 20, 1223–1226. 10.1109/LSP.2013.2285579

[B45] Smith S. M. Miller K. L. Salimi-Khorshidi G. Webster M. Beckmann C. F. Nichols T. E.. (2011). Network modelling methods for FMRI. Neuroimage 54, 875–891. 10.1016/j.neuroimage.2010.08.063 20817103

[B46] Smith S. M. Vidaurre D. Beckmann C. F. Glasser M. F. Jenkinson M. Miller K. L.. (2013). Functional connectomics from resting-state fMRI. Trends Cogn. Sci. 17, 666–682. 10.1016/j.tics.2013.09.016 24238796 PMC4004765

[B47] Sporns O. (2011). Networks of the Brain. Cambridge, MA: MIT Press.

[B48] Stam C. J. (2014). Modern network science of neurological disorders. Nature Rev. Neurosci. 15, 683–695. 10.1038/nrn3801 25186238

[B49] Supekar K. Menon V. Rubin D. Musen M. Greicius M. D. (2008). Network analysis of intrinsic functional brain connectivity in Alzheimer's Disease. PLoS Comput. Biol. 4:e1000100. 10.1371/journal.pcbi.1000100 18584043 PMC2435273

[B50] Theije C. G. M. D. Wu J. Silva S. L. D. Kamphuis P. J. Garssen J. Korte S. M.. (2011). Pathways underlying the gut-to-brain connection in autism spectrum disorders as future targets for disease management. Eur. J. Pharmacol. 668(Suppl 1.), S70–S80. 10.1016/j.ejphar.2011.07.013 21810417

[B51] Tomioka R. Sugiyama M. (2009). Dual augmented lagrangian method for efficient sparse reconstruction. IEEE Signal Process. Lett. 16, 1067–1070. 10.1109/LSP.2009.2030111

[B52] Tononi G. Sporns O. Edelman G. M. (1999). Measures of degeneracy and redundancy in biological networks. Proc. Natl. Acad. Sci. U.S.A. 96, 3257–3262. 10.1073/pnas.96.6.3257 10077671 PMC15929

[B53] Tzourio-Mazoyer N. Landeau B. Papathanassiou D. Crivello F. Etard O. Delcroix N.. (2002). Automated anatomical labeling of activations in SPM using a macroscopic anatomical parcellation of the MNI MRI single-subject brain. Neuroimage 15, 273–289. 10.1006/nimg.2001.0978 11771995

[B54] Wang K. Zhang H. Ma D. Bucan M. Glessner J. T. Abrahams B. S.. (2009). Common genetic variants on 5p14.1 associate with autism spectrum disorders. Nature 459, 528–533. 10.1038/nature07999 19404256 PMC2943511

[B55] Watts D. J. Strogatz S. H. (1998). Collective dynamics of “small-world” networks. Nature 393, 440–442. 10.1038/30918 9623998

[B56] Wee C. Y. Yap P. T. Shen D. (2016). Diagnosis of autism spectrum disorders using temporally distinct resting-state functional connectivity networks. CNS Neurosci. Ther. 22, 212–229. 10.1111/cns.12499 26821773 PMC4839002

[B57] Wee C. Y. Yap P. T. Zhang D. Denny K. Browndyke J. N. Potter G. G.. (2012). Identification of MCI individuals using structural and functional connectivity networks. Neuroimage 59, 2045–2056. 10.1016/j.neuroimage.2011.10.015 22019883 PMC3254811

[B58] Wee C. Y. Yap P. T. Zhang D. Wang L. Shen D. (2014). Group-constrained sparse fMRI connectivity modeling for mild cognitive impairment identification. Brain Struct. Funct. 219, 641–656. 10.1007/s00429-013-0524-8 23468090 PMC3710527

[B59] White J. G. Southgate E. Thomson J. N. Brenner S. (1986). The structure of the nervous system of the nematode *Caenorhabditis elegans*. Philos. Trans. R. Soc. Lond. 314, 1–340. 10.1098/rstb.1986.0056 22462104

[B60] Yan C. G. Cheung B. Kelly C. Colcombe S. Craddock R. C. Martino A. D.. (2013). A comprehensive assessment of regional variation in the impact of head micromovements on functional connectomics. Neuroimage 76, 183–201. 10.1016/j.neuroimage.2013.03.004 23499792 PMC3896129

[B61] Yu R. Zhang H. An L. Chen X. Wei Z. Shen D. (2016). Correlation-weighted sparse group representation for brain network construction in MCI classification, in Medical Image Computing and Computer-Assisted Intervention – MICCAI 2016 (Springer International Publishing). 10.1007/978-3-319-46720-7_5 PMC5477788 28642938

[B62] Yuan M. Lin Y. (2006). Model selection and estimation in regression with grouped variables. J. R. Stat. Soc. 68, 49–67. 10.1111/j.1467-9868.2005.00532.x

[B63] Zhao Y. B. (2013). New and improved conditions for uniqueness of sparsest solutions of underdetermined linear systems. Appl. Mathem. Comput. 224, 58–73. 10.1016/j.amc.2013.08.010

[B64] Zhou C. Zemanová L. Zamora G. Hilgetag C. C. Kurths J. (2006). Hierarchical organization unveiled by functional connectivity in complex brain networks. Phys. Rev. Lett. 97:238103. 10.1103/PhysRevLett.97.238103 17280251

