# Supplementary material for: Remodeling Pearson's Correlation for Functional Brain Network Estimation and Autism Spectrum Disorder Identification
Source: Front Neuroinform. 2017 Aug 31;11:55. doi: 10.3389/fninf.2017.00055 (PMC5583214; doi:10.3389/fninf.2017.00055)
Supplement: Supplementary file 1 [file Presentation1.PDF]

Supplementary Material

In order to verify the extensibility of the proposed method, we conducted experiments on a non-ASD dataset from ADNI (http://adni.loni.ucla.edu). In particular, 110 participants including 51 MCI and 59 NC participants from this dataset were adopted in this experiment. The observed fMRIs were scanned by 3.0T Philips scanners with the following parameters: TR/TE = 3000/30mm, flip angle = 80, imaging matrix=64×64, 48 slices, 140 volumes, and voxel thickness = 3.3mm. SPM8 toolbox (http://www.fil.ion.ucl.ac.uk.spm) was used to preprocess the fMRI data according to the well accepted pipeline. The first 10 R-fMRI volumes of each subject were discarded to avoid signal shaking. The remaining images were first corrected for different slice acquisition timing and head motion. Regression of ventricle and WM signals as well as six head-motion profiles were conducted to further reduce the effects of nuisance signals. Mean fMRI series of each ROI was band pass filtered (0.01-0.08Hz) for functional connectivity estimation prior. Depending on the automated anatomical labeling (AAL) atlas (Tzourio-Mazoyer et al., 2002), the pre-processed BOLD time series signals were partitioned into 116 ROIs. At last, we put these time series into a data matrix $X \in R^{137\times116}$. The setting of parameters (e.g., hard threshold in PC and λ in $PC_{sparsity}$) was same as the experiments on ABIDE dataset. The results are given in Table 5.

Table 5. Classification performance corresponding to different FBN estimation methods on ADNI dataset.

Method	Accuracy	Sensitivity	Specificity
$PC_{threshold}$	80.01	84.31	76.27
SR	68.18	72.55	64.41
$PC_{sparsity}$	82.73	78.73	86.64
$PC_{scale\text{-}free}$	**88.18**	**92.16**	**84.15**

Also, the classification accuracy corresponding to different parametric combinations was shown in in **Fig. 6**.

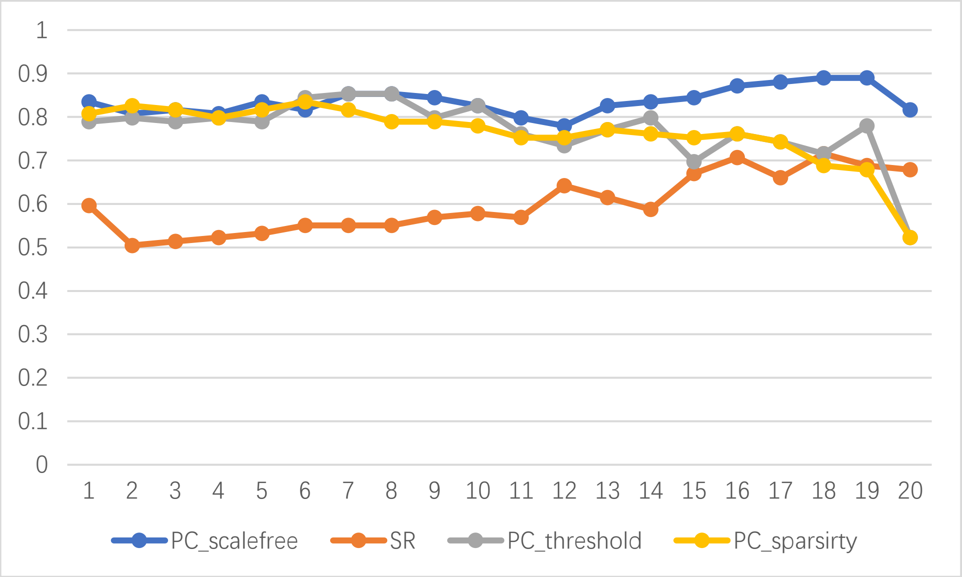

Fig.6 Classification accuracy based on the networks estimated by four different methods of 20 regularized parameters. The results are calculated by LOO test on all subjects in ADNI.
